# Impact of 5-HTTLPR on hippocampal subregional activation in older adults

**DOI:** 10.1038/tp.2015.131

**Published:** 2015-09-22

**Authors:** A Garrett, S Gupta, A L Reiss, J Waring, K Sudheimer, L Anker, N Sosa, J F Hallmayer, R O'Hara

**Affiliations:** 1 Department of Psychiatry and Behavioral Sciences, Stanford University School of Medicine, Stanford, CA, USA; 2 Center for Interdisciplinary Brain Sciences Research, Stanford University School of Medicine, Stanford, CA, USA; 3 Sierra Pacific Mental Illness, Research, Education and Clinical Center, VA Palo Alto Health Care System, Palo Alto, CA, USA

## Abstract

Studies have shown that a functional polymorphism of the serotonin transporter gene (5-HTTLPR) impacts performance on memory-related tasks and the hippocampal structures that subserve these tasks. The short (*s*) allele of 5-HTTLPR has been linked to greater susceptibility for impaired memory and smaller hippocampal volume compared to the long allele (*l*). However, previous studies have not examined the associations between 5-HTTLPR allele and activation in subregions of the hippocampus. In this study, we used functional magnetic resonance imaging (fMRI) to measure activation in hippocampal and temporal lobe subregions in 36 elderly non-clinical participants performing a face–name encoding and recognition task. Although there were no significant differences in task performance between *s* allele carriers and *l* homozygotes, right CA1 and right parahippocampal activation during recognition errors was significantly greater in individuals bearing the *s* allele. In an exploratory analysis, we determined that these effects were more pronounced in *s* allele carriers with the apolipoprotein ɛ4 allele. Our results suggest that older individuals with the *s* allele inefficiently allocate neural resources while making errors in recognizing face–name associations, which could negatively impact memory performance during more challenging tasks.

## Introduction

Cognitive and memory impairment are highly prevalent in older adults, but individual variation is substantial and the neurocircuitry subserving these impairments requires more investigation. Genetic moderators of cognitive function have been proposed as one explanation for the substantial variability in performance deficits associated with age. One such moderator is the serotonin transporter gene (5-HTTLPR), a polymorphism that is composed of a 44-base-pair insertion (*l* allele) or deletion (*s* allele). The serotonin transporter helps to regulate serotonin levels within the synapse. The *s* allele, or short form of 5-HTTLPR, is associated with reduced efficiency of transcription and reuptake of serotonin compared to the *l* allele, or long form.

The 5-HTTLPR was initially examined in the context of gene by environment interactions, showing a strong correspondence with risk for psychopathology.^1^ However, a significant literature now suggests that the *s* allele is associated with poorer cognitive performance across a broad range of cognitive domains, including impaired verbal learning and memory in both patients and controls.^2, 3, 4^ In our own investigation of healthy older adults, we found *s* allele carriers had significantly poorer performance in a dose-related fashion on a measure of delayed verbal recall.^3^ Further, we observed *s* allele carriers to have smaller hippocampal volume in the presence of physiological stress. Together, these studies implicate the 5-HTTLPR short allele in poorer memory function in addition to its role in affective processes.

However, not all studies have found the *s* allele to have deleterious effects on cognition.^5^ Indeed, healthy *s* carriers have been found to outperform *l* homozygotes on a range of cognitive domains, including executive function and working memory.^6, 7, 8^ While Roiser *et al.*^9^ observed *s* allele homozygotes to perform better overall on a measure of verbal recall than their *ll* allele counterparts, their memory performance was much more vulnerable to the negative impact of serotonin depletion.^9^ Further, it is important to note that the studies that observed a positive impact of the *s* allele, including animal investigations, included samples that were, on average, much younger than those investigations that observed a negative impact of the *s* allele. All studies of late-life subjects, on the other hand, report that *s* carriers demonstrated inferior performance across several cognitive domains, including memory.^3, 10, 11^ This suggests that the 5-HTTLPR *s* allele may negatively affect cognition in older adults, and the *l* allele may protect memory in older adults.

Another gene strongly related to impaired cognition in older adults is the apolipoprotein ɛ4 allele (APOE ɛ4). In particular, it has been consistently found to be significantly associated with impaired delayed verbal recall in older adults^12, 13^ and smaller hippocampal volume.^14, 15^ As the hippocampus is vital for successful memory encoding and retrieval,^16, 17^ the APOE allele may contribute to memory deficits in older patients with the 5-HTTLPR *s* allele.

The current study aims to increase understanding of the impact of 5-HTTLPR on hippocampal function in older adults, by examining subregional hippocampal activation during functional magnetic resonance imaging (fMRI) using a face–name encoding and recognition task in 40 healthy older adults who were selected based on 5-HTTLPR genotype. As our previous study observed a link between hippocampal volume and memory that varied by 5-HTTLPR genotype, the current study refines our understanding of this association by examining the function of individual hippocampal subfields during a recognition memory task. Based on previous reports, we hypothesized that the dentate gyrus and CA2/3 would be activated during encoding, while CA1 and subiculum would be activated during recognition.^18, 19, 20, 21, 22, 23, 24^ In an exploratory analysis, we also examined the contribution of APOE status to hippocampal activation.^25^

## Materials and methods

### Participants

Participants were 40 community-dwelling older adults participating in ongoing investigations of age-related cognitive decline at our laboratory, and selected on the basis of APOE ɛ4 status and 5-HTTLPR genotype as follows: *s* allele carrier/negative APOE ɛ4 (*n*=10); *s* allele carrier/positive APOE ɛ4 (*n*=10); *ll* homozygote/negative APOE ɛ4 (*n*=10); *ll* homozygote/positive APOE ɛ4 (*n*=10). All subjects provided informed consent for their participation in accordance with Stanford University institutional review board regulations.

The sample included 23 females and 17 males. Subjects were between 63 and 86 years of age (*M*=73.08; s.d.=5.89), with an average of 16.08 years of education (s.d.=2.1). All subjects were Caucasian and had sufficient visual and auditory acuity for cognitive testing. An initial evaluation included self-reported current and past medical status, administration of the Mini-Mental State Exam^26^ and Structured Clinical Interview for DSM-IV-TR.^27^ All subjects with a Mini-Mental State Exam score of less than 26 or any evidence of possible dementia based on their cognitive functioning or with any Axis I disorder, including any evidence of depression, were excluded.

Participants were excluded from the study if they were currently using any systemic corticosteroids, psychotropic medication, short-acting anxiolytics, sedative hypnotics, or medications with significant cholinergic or anticholinergic side effects, as well as any US Food and Drug Administration-approved medications for the treatment of Alzheimer's disease.

### Genotyping

DNA was extracted from 200 μl of frozen blood using the Qiagen DNeasy Kit (Qiagen, Valencia, CA, USA; Cat. #69506). Oligonucleotide primers flanking the 5-HTT-linked polymorphic region^28^ and corresponding to the nucleotide positions −1416 to −1397 (stpr5, 5′-GGCGTTGCCGCTCTGAATGC) and −910 to −888 (stpr3, 5′-GAGGGACTGAGCTGGACAACCAC) of the 5-HTT gene 5'-flanking regulatory region were used to generate 484 or 528 bp fragments. Polymerase chain reaction (PCR) amplification was carried out in a final volume of 30 μl consisting of 50ng of genomic DNA, 50ng each of sense and antisense primers, 15 μl of Taq PCR Master mix (Qiagen, Cat. #201445), 10% DMSO and 1M betaine. Annealing was carried out at 61 °C for 30 s, extension at 72 °C for 1 min and denaturation at 95 °C for 30 s for a total of 35 cycles. The PCR products were electrophoresed through 5% polyacrylamide gel (acrylamide/bis-acrylamide ratio 19:1) at 120 V for 60 min. A 100-bp marker was used to measure the PCR product size for *l* and *s* alleles.

APOE genotyping was performed according to the restriction isotyping protocol of Hixson and Vernier.^29^ Amplification reactions were carried out in 30 μl volume reactions containing 1 μg of DNA, 1 pmol μl^−1^ of each primer, 10% dimethyl sulfoxide, and 0.025 units μl^−1^ Taq polymerase. Following an initial denaturation step for 5 min at 95 °C amplification was achieved by 30 cycles of 60 °C for 1 min, 70 °C for 2 min and 95 °C for 1 min. After PCR amplification, 5 units of *Hha*I (New England Biolabs, Ipswich, MA, USA) were added directly to each reaction mixture for 3 h at 37 °C. Each reaction mixture was loaded onto an 8% polyacrylamide gel. Restriction digestion products were visualized on ethidium bromide staining. Two independent observers, who were blind to any information pertaining to the participants, assigned the alleles and genotypes.

### MRI data acquisition

Imaging data were acquired at the Lucas Imaging Center on a 3.0-T General Electric MR750 scanner using an eight-channel whole head coil (GE Medical Systems, Milwaukee, WI, USA). Structural images of the hippocampus and medial temporal lobe cortices were prescribed as oblique slices perpendicular to the main axis of the hippocampus, using a high-resolution T2-weighted, flow-compensated spin-echo pulse sequence with the following parameters: repetition time=4600 ms; echo time=71 ms; flip angle=90; 512 × 512 matrix; 0.43 × 0.43 mm in-plane resolution; 30 contiguous slices at 3-mm thickness per slice; 220 mm field of view. Functional images were acquired at the same slice locations, using a T2*-sensitive gradient echo spiral in/out pulse sequence and the following parameters: repetition time=2000 ms; echo time=30 ms; flip angle=77° 64x64 matrix; 3.4 × 3.4 mm in-plane resolution. A high-order shimming procedure, based on spiral acquisitions, was used to reduce B0 heterogeneity. Importantly, spiral fMRI methods increase signal-to-noise and blood oxygenation level dependent contrast-to-noise ratio while reducing signal loss in regions susceptible to field gradients, such as the medial temporal lobe.^30^

### Associative memory task

All subjects practiced an alternate version of the task before the scan to ensure that they understood and could perform the task. Thus, subjects understood that their memory for the stimuli would be tested. The task was a face/name associative memory paradigm that was previously shown to activate the hippocampus in healthy volunteers.^31^

For this task, there were four cycles of the following sequence: (1) STUDY–(2) DISTRACT–(3) TEST. During each STUDY phase, photographs of 12 young adult faces, each paired with a name printed below the photograph, were presented for 4 s each, with a 0.1- s interstimulus interval featuring a fixation cross. Subjects are instructed to learn the name associated with each face, and to press a button when each new face/name appears on the screen. For the DISTRACT phase, an instruction screen cues subjects to silently count backwards for 6 s, in order to prevent them from rehearsing the names. Finally the TEST phase presents 24 face/name pairs, 12 of which are targets and 12 are foils, presented for 4 s each. Targets are a face and name that are correctly paired according to the TEST phase. Foils showed a previously viewed face and name that are incorrectly paired. Subjects pressed button 1 if the face is paired with the correct name and button 2 if the face and name were incorrectly paired. All of the faces and names presented in the TEST phase had been presented in the previous STUDY phase, only the pairing was correct or incorrect. However, the faces and names were not repeated in subsequent STUDY–TEST cycles, for example, each STUDY–TEST cycle contained entirely new faces and names, and subjects were instructed (before the scan) to ‘forget' the previous cycle faces and names each time a new cycle began. All face photographs had ‘neutral' expressions and were taken from the McArthur stimulus set (macbrain.org/resources.htm). Half of the photographs showed male and half showed female adults. The task was presented using Eprime software, which also collected the responses. The total task time was about 10 min. As is standard practice in our laboratory, all participants were interviewed after the scan to confirm that they had performed the task according to the instructions.

### Functional MRI analyses

fMRI data were analyzed using SPM5 (Statistical Parametric Mapping version 5; Wellcome Department of Cognitive Neurology, London, UK) and associated MATLAB programs (The MathWorks, Natick, MA, USA). Pre-processing included slice timing correction, realignment to the first volume, and motion correction using ArtRepair toolbox (http://cibsr.stanford.edu/tools/human-brain-project/artrepair-software.html). The fMRI series was co-registered to the anatomical image, so that activation in subfields could be located and extracted. To preserve spatial resolution, data were not spatially smoothed or normalized, following protocols from previous studies of hippocampal subfields.^24^

Voxel-based statistical analyses were conducted at the individual participant level, using the general linear model and accounting for the intrinsic autocorrelation in fMRI data. We modeled the STUDY and TEST conditions as events within each block. Events during the STUDY block were classified as ‘correct' or ‘incorrect' based on accuracy of recognition during the subsequent TEST block. ‘STUDY correct' trials were those in which the face/name pair presented at that time was correctly recognized as a pair during the subsequent TEST Block; ‘STUDY incorrect' trials were those that were not correctly recognized during the subsequent TEST block; ‘TEST correct' trials were those receiving an accurate response during the TEST block, and ‘TEST incorrect' trials were those receiving an inaccurate response during the TEST block. Thus, each subject had a unique statistical model based on task accuracy. Each subject's model was reviewed in SPM to verify that the conditions were orthogonal and that there were a sufficient number of error and correct trials. Two subjects were rejected because of invalid statistical models: 1 subject had 98% accuracy on the task, so the incorrect trial models had too few events to be accurately estimated. The other subject was rejected for having too few correct trials to create an accurate model. Each trial was modeled as an impulse function with a duration of 4 s, convolved with a canonical hemodynamic response function. Contrasts were created for (1) ‘STUDY correct' versus ‘STUDY incorrect' and (2) ‘TEST correct' versus ‘TEST incorrect'.

### Manually traced medial temporal lobe and hippocampal subfields

**Figure 1:**
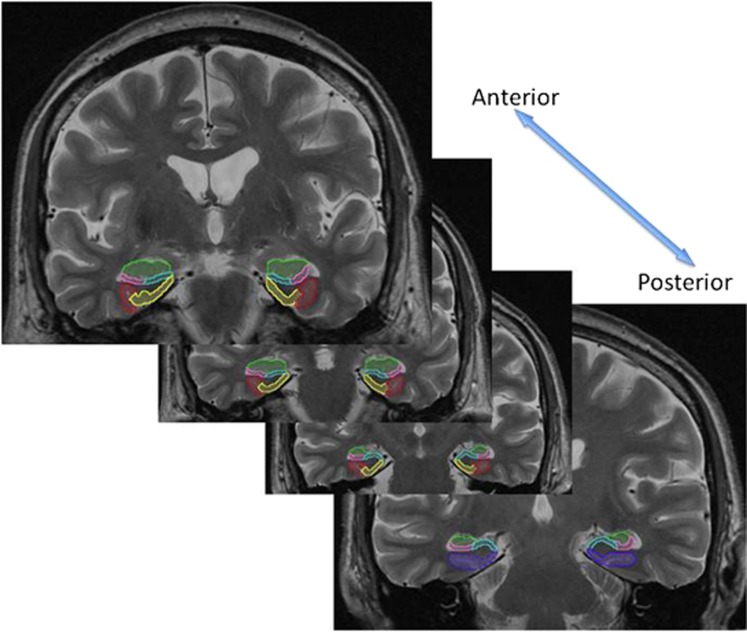
Hippocampal subregions were manually drawn on the high-resolution T2 image. Shown above, moving from anterior to posterior, the subregions included the CA1 (pink), CA2, CA3 and dentate gyrus (green), the subiculum (blue), the entorhinal cortex (yellow), the perirhinal cortex (red), and the parahippocampal gyrus (purple).

To define the boundaries of the hippocampal subregions for each subject, we traced on the native-space 512x512 T2-weighted high-resolution structural images and used previously published protocols for demarcating the subregions.^18, 32, 33, 34, 35, 36, 37^ Brain Image Java software (http://cibsr.stanford.edu/tools/human-brain-project/highlights.html) was used to trace the subregions and to measure their volumes. Before tracing regions for the study, we demonstrated a high inter-rater reliability of volumetric measurements of all subregions across two independent raters (intraclass correlation coefficient=0.85 or greater). The 12 regions included the bilateral CA2/3/dentate gyrus, CA1, subiculum, perirhinal cortex, parahippocampal cortex and entorhinal cortex. All regions of interest (ROIs) included gray and white matter, but excluded cerebrospinal fluid. The full protocol is available from the authors upon request, and subregions are illustrated in Figure 1.

Activation in each of the 12 regions was measured using the manually traced ROIs as masks on the co-registered fMRI image. Activation was quantified as the mean contrast value of all voxels in that ROI that passed a threshold of 0. A threshold of 0 was used because we were interested in activation rather than de-activation to each contrast. The mean contrast value was also weighted by the percentage of voxels in that region passing a threshold of 0. The weighted mean was used so that our measure would account for both the intensity and the extent of activation within each ROI.

### 5HT *s* allele group differences in hippocampal subregion activation and volume

Multivariate analysis of variance was implemented in SPSS software (spss.com) to test for group differences in hippocampal subregional activation. The fixed factor was allele group (*ll* versus *s*) and the dependent variables included activation in all 12 subregions, including right and left hemispheres (for example, left and right CA1, CA23DG, subiculum, perirhinal cortex, entorhinal cortex, parahippocampal cortex). Four separate models were created to test the four conditions of interest: (1) successful encoding; (2) encoding errors; (3) successful recognition; (4) recognition errors. This approach was used to test whether the profile of activation across all subregions in response to each task condition was different for each allele group. We used separate models for each of the four conditions because it is likely that there is shared variance across the task conditions. A corrected threshold of *P*=0.05 divided by four models (=0.0125) was used for the significance of each model. For each model that reached significance, we also reported the subregions contributing significantly to the model, and used follow-up *t*-tests to determine the direction of the effect. Finally, for those subregions reaching significance, we tested the moderating effect of APOE ɛ4 status using a two-way analysis of variance. For all models, age and sex were included as covariates.

## Results

### Subject characteristics

All of the subjects tolerated the scan procedure without difficulty. Data from four subjects were rejected for the following reasons: abnormal scan finding (one subject), excessive head motion (one subject) and memory task performance that did not allow us to create valid statistical models of fMRI activation (two subjects, as described above).

The final subject groups included 20 *ll* and 16 *s*. As shown in Table 1, these groups were matched on age, percentage of male subjects, number of motion artifacts in the fMRI data, memory task accuracy and response time. The average accuracy of memory task performance was 60%, with a standard deviation of 12, which allowed an adequate number of both correct (about 60) and incorrect trials (about 36) for analysis.

### Group differences in hippocampal subregion activation

**Figure 2:**
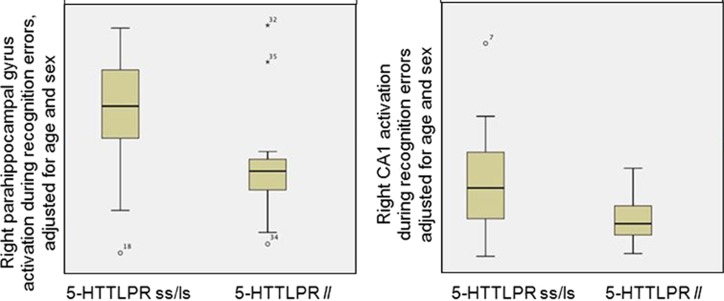
Boxplot showing signficant differences between the 5-HTTLPR allele groups in activation in the right parahippocampal gyrus (left plot) and right CA1 region (right plot). 5-HTTLPR, serotonin transporter gene; l, long allele; s, short allele.

The multivariate analysis of variance testing for allele group differences in activation during successful encoding, encoding errors and successful recognition were not significant (all F's <1). However, during recognition errors, the *s* allele group had signficantly greater activation than the *l* group across all hippocampal subregions combined (F(12,35)=4.45, *P*=0.001). This model accounted for 72% of the variance in allele group (partial Eta squared=0.718). Individual subregions contributing signficantly to this main effect included right CA1 (*P*=0.019) and right parahippocampal gyrus (*P*=0.009), in which activation during recognition errors was greater in the *s* group than in the *l* group. A boxplot showing these group differences is shown in Figure 2.

### Behavioral relevance of parahippocampal activation

Given the significant finding of greater right CA1 and right parahippocampal activation during recognition errors in the *s* allele group, we investigated task performance correlates of activation in this region to help interpet its meaning. We performed a Pearson's correlation between right parahippocampal activation and task accuracy. The correlation was not significant across allele groups (*r*(35)=0.21, *P*=0.22), nor within each allele group separately (*s* group: *r*(35)=0.15, *P*=0.53; *l* group: *r*(35)=0.28, *P*=0.29). Similarly, for right CA1, activation was not correlated with task accuracy across allele groups (*r*(35)=0.14, *P*=0.42) nor within allele groups (*s* group: *r*(35)=0.27, *P*=0.26; *l* group: *r*(35)=−0.21, *P*=0.44).

### Exploratory analysis of the moderating effect of apolipoprotein E

Given that our main analysis showed that activation in the right CA1 and right parahippocampal gyrus during recognition errors distinguishes the *s* group from the *l* group, we ran an exploratory analysis of the moderating effect of APOE ɛ4 allele. We used a 2 × 2 analysis of variance with activation as dependent variable and 5-HTTLPR group (*l* versus *s*) and APOE ɛ4 allele carrier status as the independent variables. Age and sex were covaried. This analysis should be considered preliminary, as the sample size of the subgroups is small when considering the effects of both genotypes. For the right CA1, the overall model was significant (F(5,35=3.05, *P*=0.02), and so was the main effect of the 5-HTTLPR group (F(1,35)=6.03, *P*=0.02), but the main effect of the APOE group (F(1,35)=3.52, *P*=0.07) and the interaction (F<1) were not significant. The overall model for the right parahippocampal cortex was significant (F(5,35=4.02, *P*=0.004), and so were the main effect of the 5-HTTLPR group (F(1,35)=7.59, *P*=0.01) and the main effect of the APOE group (F(1,35)=8.72, *P*=0.006), with activation being higher in those positive for APOE ɛ4. The interaction was not significant (F<1).

### Group differences in subregion volumes

The allele groups did not differ in volume of any of the 12 ROIs, with or without controlling for total brain volume (Table 2). Although we did not have hypotheses about volumetric differences between groups, this comparison helped to confirm that any group differences in activation were not attributed to differences in volume. Although exploratory, we also investigated interactions between the allele group and APOE group on subregional volume. Only the volumes of the right subiculum showed an interaction between 5HTTLPR *s* allele and APOE ɛ4 allele (F(1,36)=4.87, *P*=0.035) that did not not survive correction for multiple comparisons (corrected p=0.05/12=0.004). For this trend, participants with the *l* allele who had the ɛ4 allele had larger volumes than *l* allele carriers without the ɛ4 allele, while in the *s* group, APOE allele had no effect on volume.

## Discussion

In this study, we observed that elderly participants with the *s* allele, compared to those with the *ll* allele of the 5-HTTLPR gene, had signficantly different subregional hippocampal activation during a face–name recognition task. Among the four task conditions (successful encoding, encoding errors, successful recognition and recognition errors), differential activation was observed by 5-HTTLPR genotype only for recognition errors. Specifically, *s* allele carriers exhibited greater activation of the right parahippocampal and right CA1 regions during recognition errors, compared to *l* allele homozygotes, despite similar task accuracy. Furthermore, activation was greatest in *s* allele carriers who were positive for the APOE ɛ4 allele, consistent with previous reports that young adult APOE ɛ4 carriers have greater hippocampal activation than noncarriers when performing a memory task.^38^

Given the lack of group differences in task accuracy and response time, greater activation observed in the *s* allele and ɛ4 positive groups suggests neural compensation for inefficient processing in the parahippocampal and CA1 regions. This hypothesis of compensatory neuronal activity has long been conjectured to explain greater activation in older compared to young adults during similar task performance,^39^ and compensatory activation has been reported in older ɛ4 carriers during a fMRI memory paradigm.^40, 41^ However, the association between activation and memory accuracy is not clear. While one study has reported that increasing activation is correlated with improved memory performance among elderly adults,^42^ another study found that greater parahippocampal activation is associated with false rather than accurate retrieval of information.^43^ In the current study, we found no correlation between activation and memory accuracy. Another interpretation of our finding of greater activation during recognition errors is that individuals with the *s* allele and/or APOE ɛ4 are less certain that they recognize the name or face, and therefore are expending more effort when performing the task. Indeed, there is a significant literature to suggest that older adults are more likely to commit errors of recognition.^44^

The hippocampus and parahippocampal gyrus are vital for successful memory encoding and retrieval,^16, 17^ and subregions of the hippocampus may be specialized for different facets of memory. The CA1 region has been implicated in forming associations over time and is important for recognition memory,^17, 45^ consistent with our findings. The parahippocampal gyrus is important for remembering contextual information,^16^ which is critical for our associative memory task. Regarding our observation of group differences in the right, and not left hemisphere, this finding is in line with previous studies finding predominantly right hippocampal activation during memory tasks requiring subjects to make relational attributions, as is the case in our face–name associative recognition task.^46, 47^ Recent imaging studies suggest that the hippocampus also may be involved in visual processing.^48^ As such increased activation of these regions in our investigation may reflect not only memory processing, but also the ability to process and discriminate among the complex facial stimuli, greater activation may reflect compensatory mechanisms or greater effort during both memory and perceptual processes.

The limitations of our investigation include the small sample size, particularly for examining the interactive effects of 5-HTTLPR and APOE genotypes. This may have reduced our power for detecting group differences in task accuracy as well. Additionally, we focused only on the hippocampal area so that we could investigate subregional activation. Future studies could examine other brain regions that may be impacted by 5-HTTLPR genotype, including the anterior cingulate, amygdala and prefrontal cortex. Additionally, we selected participants who were not depressed. Given the role of 5-HTTLPR in moderating depressive symptoms, future investigations may want to consider whether the observed effects contribute to the impaired memory function that is frequently observed in older adults with depression.

Our findings underscore the need to consider the role of the 5-HTTLPR polymorphism in the context of other genetically determined processes that impact brain and cognition. Although our findings were observed in healthy older adults, they have implications for both depression and cognitive impairment in late life, and the potential interaction. For example, Geda *et al.*^49^ observed a significant interaction of the APOE ɛ4 allele and onset of depression in the development of MCI and progression to dementia, supporting the view that impaired serotonergic function may also contribute to the development of dementia in those at higher risk for the illness. Further, smaller hippocampal volumes predict slower response to antidepressant treatment in late life depression,^50^ and many investigators have suggested that there is reciprocal relationship between hippocampal volume, depression and cognitive function in the elderly.^51, 52, 53, 54^ The role of depression in the development of MCI and dementia may reflect interactive effects of the 5-HTTLPR *s* allele and presence of the ɛ4 allele. This speculation is in line with the suggestion of Smith *et al.*^55^ that 5-HTT genotype may impact the normal aging process, in terms of reduced capacity for older adults with the *s* allele to adapt to age-related alterations in serotonin function, with the resulting emergence of behavioral symptoms, particularly secondary to neurodegenerative diseases. Future investigations are needed to determine whether the 5-HTTLPR *s* allele and APOE ɛ4 allele interact to contribute to impaired memory function in older adults with depression.

In sum, our results suggest that older individuals with the 5-HTTLPR *s* allele may either inefficiently allocate neural resources while making errors in recognizing face–name associations, or compensate with greater activation to accomplish the memory task. These effects appear to be more extreme in *s* allele carriers who also are positive for the apolipoprotein ɛ4 allele. As such, *s* allele carriers, positive for the ɛ4 allele, may be at particular risk for increased memory performance difficulties with age, particularly during more challenging memory tasks.

## Figures and Tables

**Table 1:** Comparison of 5-HTTLPR group characteristics

*Measure*	*5-HTTLPR genotype*		P
	*ll Group (*N=*16)*, *mean (s.d.)*	*ss/sl Group (*N=*20),* *mean (s.d.)*	
Age	73.85 (5.20), range=63–84	72.30 (6.58), range=64–86	0.43
			
% Male	44%	50%	0.72
			
# Motion artifacts during fMRI	11.20 (13.9)	16.30 (16.84)	0.33
			
fMRI face–name memory task: accuracy of recognition (percent correct)	61.13 (9.09), range=42.71–75.00	62.14 (8.27), range=47.92–75.00	0.73
			
fMRI face–name memory task: response time during recognition trials (ms)	2036.86 (285.74), range=1197–2364	2005.84 (254.20), range=1556–2574 ms	0.74

Abbreviations: fMRI, functional magnetic resonance imaging; 5-HTTLPR, serotonin transporter gene; *l*, long allele; *s*, short allele; s.d., standard deviation.

**Table 2:** Volumes of hippocampal and medial temporal cortical regions of interest per 5-HTTLPR allele group

*Brain regional volume (mm*^*3*^)	*5-HTTLPR genotype*		P
	*ll Group (*N=*16),* *mean (s.d.)*	*ss/sl Group (*N=*20)*, *mean (s.d.)*	
Total brain volume	1146.52 (92.09)	1152.83 (120.64)	0.86
Right perirhinal cortex	0.0302 (0.0087)	0.0317 (0.0084)	0.61
Left perirhinal cortex	0.0327 (0.0072)	0.0356 (0.0107)	0.33
Right entorhinal cortex	0.0224 (0.0069)	0.0222 (0.0056)	0.95
Left entorhinal cortex	0.0205 (0.0041)	0.0227 (0.0053)	0.17
Right parahippocampal gyrus	0.0351 (0.0092)	0.0322 (0.0045)	0.26
Left parahippocampal gyrus	0.0346 (0.0067)	0.0334 (0.0077)	0.62
Right CA1 subregion	0.0103 (0.0028)	0.0099 (0.0017)	0.71
Left CA1 subregion	0.0087 (0.0019)	0.0088 (0.0019)	0.95
Right CA23DG subregion	0.0303 (0.0091)	0.0312 (0.0062)	0.73
Left CA23DG subregion	0.0263 (0.0064)	0.0273 (0.0062)	0.66
Right subiculum subregion	0.0116 (0.0029)	0.0110 (0.0021)	0.50
Left subiculum subregion	0.0116 (0.0026)	0.0109 (0.0025)	0.40

Abbreviations: DG, dentate gyrus; 5-HTTLPR, serotonin transporter gene; *l*, long allele; *s*, short allele; s.d., standard deviation. CA1 and CA23DG indicate subregions of the hippocampus.
